# ER bodies in plants of the *Brassicales* order: biogenesis and association with innate immunity

**DOI:** 10.3389/fpls.2014.00073

**Published:** 2014-03-10

**Authors:** Ryohei T. Nakano, Kenji Yamada, Paweł Bednarek, Mikio Nishimura, Ikuko Hara-Nishimura

**Affiliations:** ^1^Department of Plant Microbe Interactions, Max Planck Institute for Plant Breeding ResearchCologne, Germany; ^2^Department of Cell Biology, National Institute for Basic BiologyOkazaki, Japan; ^3^Department of Basic Biology, School of Life Science, The Graduate University for Advanced Studies (Sokendai)Okazaki, Japan; ^4^Institute of Bioorganic Chemistry, Polish Academy of SciencesPoznañ, Poland; ^5^Department of Botany, Graduate School of Science, Kyoto UniversityKyoto, Japan

**Keywords:** endoplasmic reticulum, ER body, organelle biogenesis, β-glucosidase, plant defenses, secondary metabolites, glucosinolate

## Abstract

The endoplasmic reticulum (ER) forms highly organized network structures composed of tubules and cisternae. Many plant species develop additional ER-derived structures, most of which are specific for certain groups of species. In particular, a rod-shaped structure designated as the ER body is produced by plants of the *Brassicales* order, which includes *Arabidopsis thaliana*. Genetic analyses and characterization of *A. thaliana* mutants possessing a disorganized ER morphology or lacking ER bodies have provided insights into the highly organized mechanisms responsible for the formation of these unique ER structures. The accumulation of proteins specific for the ER body within the ER plays an important role in the formation of ER bodies. However, a mutant that exhibits morphological defects of both the ER and ER bodies has not been identified. This suggests that plants in the *Brassicales* order have evolved novel mechanisms for the development of this unique organelle, which are distinct from those used to maintain generic ER structures. In *A. thaliana*, ER bodies are ubiquitous in seedlings and roots, but rare in rosette leaves. Wounding of rosette leaves induces *de novo* formation of ER bodies, suggesting that these structures are associated with resistance against pathogens and/or herbivores. ER bodies accumulate a large amount of β-glucosidases, which can produce substances that potentially protect against invading pests. Biochemical studies have determined that the enzymatic activities of these β-glucosidases are enhanced during cell collapse. These results suggest that ER bodies are involved in plant immunity, although there is no direct evidence of this. In this review, we provide recent perspectives of ER and ER body formation in *A. thaliana*, and discuss clues for the functions of ER bodies. We highlight defense strategies against biotic stress that are unique for the *Brassicales* order, and discuss how ER structures could contribute to these strategies.

## Introduction

The endoplasmic reticulum (ER) forms highly organized network structures composed of ER tubules and ER cisternae. In addition to this well-conserved ER network, different plant species develop unique ER-derived compartments that can be regarded as ER domains. Many of the ER-derived compartments accumulate specific types of proteins, such as the protein bodies (PBs) in maize and rice, which contain prolamin and zein, respectively (Herman and Larkins, 1999), the KDEL-tailed protease-accumulating vesicles (KVs) in mungbean (Toyooka et al., 2000), and the ricinosomes in castor bean that accumulate papain-type proteases (Schmid et al., 2001). These structures are thought to function as repositories of particular proteins until they are required.

In this review, we focus on what is called the ER body, also known as a fusiform body. This structure is an ER domain of unique shape and taxonomic distribution. In 1965, the ER body was first discovered in root epidermal and cortical cells of radish (Bonnett and Newcomb, 1965). It was described as dilated cisternae that had luminal continuity to the ER. During the following 15 years, researchers tried to characterize these structures and determine their functions (Iversen and Flood, 1969; Iversen, 1970a; Cresti et al., 1974; Hoefert, 1975; Endress and Sjolund, 1976; Jørgensen et al., 1977; Behnke and Eschlbeck, 1978; Gailhofer et al., 1979; Jørgensen, 1981). Three independent studies revealed that the dilated cisternae were primarily restricted to species of the order *Brassicales*, which were known to produce thioglucosides named glucosinolates (Iversen, 1970a; Behnke and Eschlbeck, 1978; Jørgensen, 1981). Given that glucosinolates required a specific enzyme called myrosinase to become active, this correlation implied that the dilated cisternae could act as a myrosinase repository. Indeed, activity-labeled transmission electron microscopy revealed that the dilated cisternae contained potential myrosinase activity toward sinigrin (Iversen, 1970b), although this experimental system was subsequently questioned (Behnke and Eschlbeck, 1978). The question as to whether the dilated cisternae were involved in glucosinolate metabolism was left unsolved for decades, possibly due to a lack of appropriate molecular tools. In 1998, after several analyses using the model plant *A. thaliana*, it was suggested that the rod-shaped structures labeled with ER-localized green fluorescent protein (GFP) were equivalent to the dilated cisternae (Gunning, 1998). Hayashi et al. (2001) showed that these rod-shaped structures resembled the dilated cisternae described in past literature, and designated them as ER bodies. Matsushima et al. (2004) isolated the *nai1* mutant that lacked ER bodies (“nai” is a Japanese word for “absence”), which was the very first identification of genetic material in ER body research. Since then, genetic and biochemical studies have provided insights into the functions, importance, and biogenesis of this unique organelle, which will be summarized in this review.

Although most of ER-derived compartments form spherical structures, ER bodies have a rod shape. The constitutive presence of ER bodies in *A. thaliana* is strictly limited to roots in adult plants; they are absent in most cells in rosette leaves. Both wounding and jasmonic acid treatment induce *de novo* formation of ER bodies in rosette leaves. In combination with their unique shape, one can presume a tightly regulated and unique mechanism for ER body formation, which is discussed in the first section. The tissue specificity and the inducibility also suggest that ER bodies are involved in the response to wounding and some biotic stresses, as discussed in the second section. In the last section, we will discuss how ER bodies are conserved and evolved in the plant kingdom. Current research indicates that ER bodies are specific to the order *Brassicales*, especially to the families *Brassicaceae, Capparaceae*, and *Cleomaceae*. It is known that *Brassicales* plants have unique defense strategies against biotic stresses, which may lead to an interesting evolutionary story that includes ER bodies.

## Molecular mechanisms underlying ER body formation

### ER body is a subdomain of ER that has specific organization mechanism

**Figure 1:**
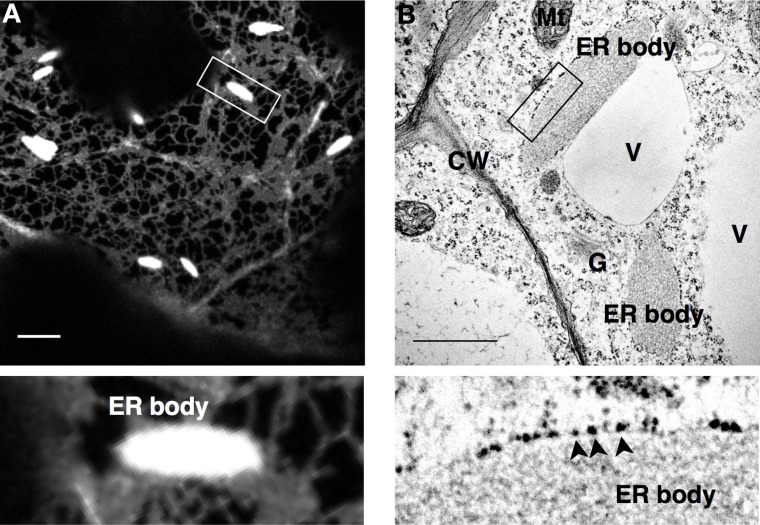
**ER bodies in *Arabidopsis thaliana***. A confocal micrograph **(A)** and an electron micrograph **(B)** of cotyledon and root epidermal cells, respectively, of *A. thaliana*. Arrowheads indicate ribosomes on the surface of the ER body membranes. ER-localized GFP (SP-GFP-HDEL) labels ER bodies as well as the typical ER network, and electron microscopy identifies ribosomes at the cytosolic surface of ER bodies, both of which indicate the luminal continuity between ER and ER bodies. Enlarged images of the squared regions are shown below. CW, cell wall; V, vacuole; Mt, mitochondrion; G, Gold body; Bars, 10 μm **(A)** and 1 μm **(B)**.

The ER body in *A. thaliana* was first observed in a transgenic line (GFP-h) expressing ER-targeted GFP. In the cotyledons of GFP-h, bright rod-shaped structures of ER bodies were observed in addition to the generic ER network (Figure 1A; Ridge et al., 1999; Hawes et al., 2001; Hayashi et al., 2001). Electron microscopy analysis revealed that the ER body is covered by a single membrane surrounded by ribosomes, which is a characteristic of the ER (Figure 1B). The ER body is observed as a structure that is connected with ER tubules and/or ER cisternae in electron micrographs (Gunning, 1998; Hayashi et al., 2001). These results indicate that the ER body is continuous to the whole ER network; therefore, it is suggested to be a subdomain of the ER that has specific functions.

Plants develop several ER-derived structures that are thought to specifically function for protein storage in specific organs or during specific life stages. These include PBs and precursor-accumulating (PAC) vesicles in maturating seeds, KVs and ricinosomes in germinating seeds, and ER bodies in roots and seedlings. PBs accumulate seed-storage proteins in monocot plants such as rice (*Oryza sativa*) and maize (*Zea mays*) (Herman and Larkins, 1999). PAC vesicles accumulate precursors of seed-storage proteins in dicot plants such as pumpkin (*Cucubita maxima*) and castor bean (*Ricinus communis*), which undergo bulk transport to the protein-storage vacuole (Hara-Nishimura et al., 1998). KVs and ricinosomes accumulate papain-type proteases for the degradation of seed-storage materials in the cotyledon of mungbean (*Vigna mungo*) or endosperm of castor bean, respectively (Toyooka et al., 2000; Schmid et al., 2001). These vesicles are spherical with diameters of 0.5–1.0 μm and are surrounded by ribosomes, indicating that they are derived from the ER. Compared to these ER-derived vesicles, the ER body is longer and larger (~10 μm long and ~1 μm wide), and accumulates different kinds of proteins, namely the β-glucosidases. Therefore, the ER body is presumably different both in function and in biogenesis mechanisms from other ER-derived structures.

### β-glucosidase is the major component of ER body

The major protein component of ER body in *A. thaliana* is identified as a β-glucosidase called PYK10/BGLU23 (Matsushima et al., 2003). This β-glucosidase was identified by analysis of the *nai1* mutant in which ER bodies were absent. PYK10 is actively recruited from the ER network to ER bodies (Matsushima et al., 2003), in contrast to that for generic ER luminal proteins such as SP-GFP-HDEL. The level of PYK10 protein is high enough to be detected as one of the most abundant proteins in *A. thaliana* roots (Matsushima et al., 2003). Electron microscopy analysis revealed a relatively high electron density in the ER body lumen, suggesting that the ER body contains a large amount of proteins (Gunning, 1998; Hayashi et al., 2001). Consistent with this, ER bodies are enriched in a relatively heavier fraction after a centrifugation-based fractionation of subcellular compartments (Matsushima et al., 2003), which confirms a high protein density within ER bodies. These studies of *A. thaliana* suggest that the ER body is a vessel for β-glucosidases to separate them from other proteins and substrates. Indeed, when cells are disrupted, PYK10 forms a huge protein complex that contains proteins originating from various subcellular compartments (Nagano et al., 2005, 2008). The *A. thaliana* genome encodes 47 β-glucosidases within 10 subfamilies (Xu et al., 2004), most of which (40 out of 47) possess signal peptides at their amino-termini, indicating localization in the endomembrane system or secretion to the apoplast (Table 1). All members of the subfamily that includes PYK10 and additional 7 β-glucosidases (BGLU18−25) also contain ER retention signals ([K/H/R][D/E]EL) at their carboxy-termini. Based on their sequence similarity, it can be assumed that these 8 β-glucosidases localize in ER bodies. Consistent with this, the subfamily member BGLU18 accumulates in ER bodies that are induced by wounding in rosette leaves (Ogasawara et al., 2009).

**Table 1:** **A summary of 47 β-glucosidases encoded in the *A. thaliana* genome**.

**Subfamily^§^**	**Alias**	**Locus**	**Signal peptide^†^**	**ER retention**	**Proton donor^‡^**	**Proton acceptor^‡^**	**Aglycone binding^‡^**	**Expression^¶^**	**Notes**
1	BGLU1	At1g45191	+	−	E	E	G	−	−
	BGLU2	At5g16580	−	−	E	E	G	−	
	BGLU3	At4g22100	+	−	E	E	G	Maturing seeds (late)	−
	BGLU4	At1g60090	+	−	E	E	G	Very low in all tissues	−
	BGLU5	At1g60260	+	−	E	E	G	−	−
	BGLU6	At1g60270	+	−	E	E	G	Aerial tissues except for reproductive tissues	−
	BGLU7	At3g62740	+	−	E	E	G	Seedlings and roots	−
	BGLU8	At3g62750	+	−	E	E	A	−	−
	BGLU9	At4g27820	+	−	E	E	G	Ubiquitous	−
	BGLU10	At4g27830	+	−	E	E	G	Ubiquitous	−
	BGLU11	At1g02850	+	−	E	E	G	Reproductive tissues and roots	−
2	BGLU12	At5g42260	+	−	E	E	Q	−	−
	BGLU13	At5g44640	+	−	E	E	Q	−	−
	BGLU14	At2g25630	+	−	E	−	Q	Pollen	−
	BGLU15	At2g44450	+	−	E	E	Q	Maturing seeds (early), flowers, and roots	−
	BGLU16	At3g60130	+	−	E	E	E	Ubiquitous	−
	BGLU17	At2g44480	−	−	E	E	L	Maturing seeds (late) and roots	−
3	BGLU18	At1g52400	+	**+**	E	E	A	Very high; aerial tissues	Induced by wounding; involved in ABA signaling
	BGLU19	At3g21370	+	**+**	E	E	S	Maturing seeds (late)	−
	BGLU20	At1g75940	+	**+**	E	E	A	Flowers	−
	BGLU21	At1g66270	+	**+**	E	E	A	−	−
	BGLU22	At1g66280	+	**+**	E	E	A	−	−
	BGLU23/PYK10	At3g09260	+	**+**	E	E	A	Seedlings and roots	ER body component
	BGLU24	At5g28510	+	**+**	E	E	A	Very low in all tissues	−
	BGLU25/GLUC	At3g03640	+	**+**	E	G	G	Ubiquitous	−
4	BGLU26/PEN2	At2g44490	−	−	E	E	A	Ubiquitous, except for maturing seeds	Atypical myrosinase; required for resistance against powdery mildew
	BGLU27	At3g60120	−	−	E	E	G	Very low in all tissues	−
5	BGLU28	At2g44460	+	−	E	E	A	Weakly in reproductive tissues and roots	−
	BGLU29	At2g44470	+	−	E	E	A	Maturing seeds	−
	BGLU30/DIN2/SRG2	At3g60140	+	−	E	E	A	Maturing seeds and roots	−
	BGLU31	At5g24540	+	−	E	E	S	Very low in all tissues	
	BGLU32	At5g24550	+	−	E	E	S	Very low in all tissues	
6	BGLU33	At2g32860	+	−	E	E	A	Ubiquitous in vegetative tissues	Involved in ABA signaling
7	BGLU34/TGG4	At1g47600	+	−	Q	E	K	−	Myrosinase in roots; resistance against insects?
	BGLU35/TGG5	At1g51470	+	−	Q	E	K	−	Myrosinase in roots; resistance against insects?
	BGLU36/TGG6	At1g51490	−	−	Q	P	K	Pollen	Myrosinase; pseudogene
	BGLU37/TGG2	At5g25980	+	−	Q	E	R	−	Myrosinase in leaves; resistance against insects?
	BGLU38/TGG1	At5g26000	+	−	Q	E	R	−	Myrosinase in leaves; resistance against insects?
	BGLU39/TGG3	At5g48375	+	−	Q	E	R	−	Myrosinase; pseudogene
8	BGLU40	At1g26560	+	−	E	E	Q	Ubiquitous except for roots	−
	BGLU41	At5g54570	+	−	E	E	Q	Reproductive tissues	−
	BGLU42	At5g36890	−	−	E	E	N	Ubiquitous except for maturing seeds	−
9	BGLU43	At3g18070	+	−	E	E	L	Very low in all tissues	−
	BGLU44	At3g18080	+	−	E	E	L	Ubiquitous	−
10	BGLU45	At1g61810	+	−	E	E	L	Stems and maturing seeds	−
	BGLU46	At1g61820	+	−	E	E	L	Maturing seeds and roots	−
	BGLU47	At4g21760	−	−	E	E	L	Leaves	−

§ *Xu et al., 2004*.

† *SignalP (http://www.cbs.dtu.dk/services/SignalP/)*.

‡ *Burmeister et al., 1997*.

¶ *ATTED-II (http://atted.jp/)*.

### NAI2 and PYK10: ER body components required for ER body formation

The protein factors involved in ER body formation in *A. thaliana* have been identified by analysis of the two mutants *nai2* and *long ER body* (*leb*). The recessive *nai2* mutant lacks ER bodies in seedlings and roots (Yamada et al., 2008). NAI2 accumulates in ER bodies but not in the ER network, indicating that NAI2 is an ER body component that determines ER body formation in *A. thaliana*. In the absence of NAI2, PYK10 is diffused throughout the ER network, and the protein levels are lower compared to those for wild type (WT). These results show that NAI2 enables the high accumulation and storage of PYK10, presumably by mediating production of the ER body. Based on sequence similarity, NAI2 homologs are only observed in *Brassicaceae* plants that produce ER bodies, further suggesting that NAI2 has a specific function in the generation of this organelle.

In *leb* mutant seedlings, there are fewer ER bodies and they have an elongated shape compared to those in WT seedlings (Nagano et al., 2009). A mutation in the *PYK10* gene that causes an amino acid substitution is responsible for the *leb* phenotype, revealing the importance of PYK10 localization in the ER body for ER body formation. PYK10 forms an oligomer linked by a disulfide bond (Nagano et al., 2005), presumably via a cysteine residue that is substituted in the *leb* mutant, because the mutation resulted in an altered oligomeric structure and reduced accumulation of PYK10 protein (Nagano et al., 2009). The *pyk10* single knockout mutant exhibited milder phenotype than that of the *leb* mutant, indicating that the *leb* mutation affects other ER body components that contribute to proper ER body organization. BGLU21 and BGLU22 are the two closest homologs of PYK10, which might contribute to ER body formation. A double knockout mutant *pyk10 bglu21* showed similar phenotype to the *leb* mutant, indicating that BGLU21 is involved in ER body formation. The *PYK10* mutation affects the nature of BGLU21 protein such as oligomeric states and/or protein conformation and induces the formation of aberrant ER bodies. These data suggest that PYK10, BGLU21, and perhaps BGLU22 are redundantly important for proper organization of this organelle in *A. thaliana* seedlings. The redundancy between BGLU21 and BGLU22 is also suggested by the fact that these two proteins are more similar to each other than to the closest homolog in *Arabidopsis lyrata*.

### ER body membrane contains specific proteins

The membrane of ER bodies may have an important role in mediating the function and/or formation of ER bodies. Two integral membrane proteins, designated as MEMBRANE OF ER BODY (MEB) 1 and MEB2, have been identified to accumulate specifically at ER body membranes in *A. thaliana* (Yamada et al., 2013). These proteins were identified by coexpression analysis based on a public microarray database (ATTED-II; http://atted.jp) and transcriptomic analysis using the *nai1* mutant. MEB1 and MEB2 are homologous to each other with multiple membrane-spanning regions. They have weak similarity to Ccc1p and VIT1, an iron/manganese transporter in *S. cerevisiae* and *A. thaliana*, respectively (Li et al., 2001; Kim et al., 2006). MEB1 and MEB2 appear to possess metal transporter activity (discussed below), although their physiological role for plant fitness is still unknown (Yamada et al., 2013). MEB1 and MEB2 form a protein complex with NAI2, and are diffused throughout the ER network in the *nai2* mutant, suggesting the NAI2-dependent recruitment of MEB1 and MEB2 into ER body membranes. ER bodies in the seedlings of the *meb1 meb2* double mutant exhibited a comparable number and shape to those in WT seedlings, suggesting that these proteins are not necessary for ER body formation. However, these results indicate that the ER body membrane has a specific composition of proteins that differs from that of the ER network, and suggest that NAI2 is responsible for the organization of these ER body membrane proteins. Because NAI2 alone regulates ER body formation, the NAI2-dependent specification of the membrane by gathering specific proteins may have a crucial role for the biogenesis of ER bodies.

### NAI1: the transcription factor for ER body formation

**Figure 2:**
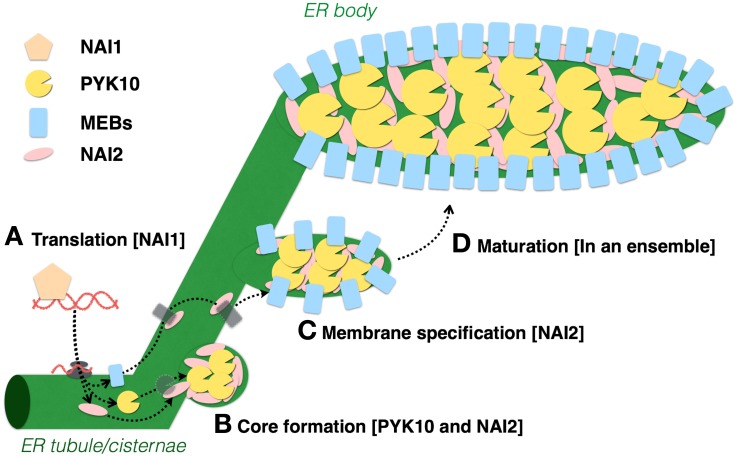
**Models of ER body formation in *A. thaliana* seedlings**. **(A)** *NAI1* is expressed in epidermal cells to regulate ER body formation by inducing the expression of *PYK10, NAI2, MEB1*, and *MEB2*. **(B)** PYK10 and NAI2 may physically interact to form ER body structures from the ER. **(C,D)** NAI2 forms a complex with MEB1and MEB2, then recruits them to the ER body to form the ER body-specific membrane.

The basic helix-loop-helix- (bHLH) type transcription factor NAI1 (also known as AtbHLH20) solely regulates ER body formation, because disruption of this gene in *A. thaliana* completely disrupts ER body formation in seedlings and roots (Matsushima et al., 2004). A transcriptomic analysis of the *nai1* mutant revealed that ER body proteins, such as those encoded by *PYK10, NAI2, MEB1*, and *MEB2*, are expressed in a NAI1-dependent manner. This indicates that NAI1 is the master regulator for ER body formation, and it regulates the expression of these genes. NAI1 also regulates the expression of *JACALIN-RELATED LECTIN* genes (*JAL22, JAL23, JAL31*, and *PYK10 BINDING PROTEIN 1* (*PBP1*)/(*JAL30*), *GDSL LIPASE-LIKE PROTEIN* genes (*GLL23* and *GLL25*), and *MD2-RELATED LIPID RECOGNITION PROTEIN 3* (*ML3*) (Nagano et al., 2005, 2008; Hakenjos et al., 2013). JAL proteins lack signal peptides and are assumed to localize in the cytosol (Nagano et al., 2005), whereas GLL25 and ML3 have signal peptides and accumulate in vacuoles (Nakano et al., 2012; Hakenjos et al., 2013). JAL and GLL proteins form a large protein complex with PYK10 when cells are collapsed or disrupted (Nagano et al., 2005, 2008; Ahn et al., 2010), suggesting their functional link to PYK10 and ER bodies. Current research indicates that there are no morphological disorders of ER bodies when these proteins are depleted, indicating their lack of importance in ER body formation. Figure 2 shows a proposed model of how the proteins are coordinated during ER body formation. Because a defect of either NAI2 or PYK10 causes disorganized ER body formation, these proteins are important for shaping ER bodies. It is possible that these proteins interact with each other to condense materials for ER bodies, followed by the NAI2-dependent recruitment of MEB1 and MEB2 to the pre-ER body membrane to form the ER bodies.

### Relationship of ER network formation and ER body formation

The ER body is derived from the ER network, and some ER proteins also localize in ER bodies, and thus it is possible that the organization of ER and ER bodies is coregulated by similar molecular mechanisms. Understanding how ER networks are formed and maintained provides insights into ER body organization. The molecular basis underlying ER morphology has been intensely studied for more than a decade using a variety of eukaryotic cells including mammals, yeasts, and plants such as *A. thaliana* and *Nicotiana benthamiana* (reviewed in Chen et al., 2013). It has been shown that ER network structures are determined by a series of membrane proteins [i.e., tubule-associated proteins such as reticulon family proteins (RTNs), Atlastin/ROOT HAIR DEFECTIVE 3 (RHD3) GTPases, and cisternae-associated proteins including CLIMP-63] (Nziengui et al., 2007; Sparkes et al., 2010; Tolley et al., 2010; Chen et al., 2011; Lee et al., 2011, 2013; Stefano et al., 2011; Zhang et al., 2013). Most studies of RTNs and Atlastin/RHD3 have been carried out using animal cells or *N. benthamiana*, which does not generate ER bodies. Therefore, it remains unclear how the plants developing ER bodies utilize these conserved mechanisms to generate these unique structures. Because reticulons and other similar proteins prefer localizing to the high curvature of ER membranes to retain a tubular structure, it is possible that these proteins are excluded from the ER bodies that have lower membrane curvature compared to that of the tubules. Alternatively, they might interact with ER body-specific proteins such as MEB1 and MEB2 on the ER body membranes, and contribute to its unique curvatures. In this context, the localization of RTNs and RHD3 in *A. thaliana* is of great interest.

In *A. thaliana*, a series of mutants designated as *endoplasmic reticulum morphology* (*ermo*) mutants have been isolated in a forward-genetic screen to identify the factors responsible for maintaining ER morphology (Nakano et al., 2009, 2012). In the *ermo1* and *ermo2* mutants, the cells develop a number of ER-derived spherical bodies, ~1 μm in diameter, in addition to the typical ER network, whereas *ermo3* cells develop huge aberrant aggregate structures derived from the ER network and ER bodies. *ERMO1* and *ERMO2* encode *GNOM-LIKE 1* (*GNL1*) and *SEC24a*, respectively, which are involved in protein transport between ER and Golgi bodies. The unaffected morphology of ER bodies suggested that both *GNL1* and *SEC24a* were dispensable for the proper organization of ER bodies (Faso et al., 2009; Nakano et al., 2009). *ERMO3* encodes a GDSL lipase-like protein also known as MODIFIED VACUOLE PHENOTYPE1 (MVP1) or GOLGI DEFECTIVE36 (GOLD36), which localizes in vacuoles (Agee et al., 2010; Marti et al., 2010; Nakano et al., 2012). The ER aggregations in *ermo3* are absent in cells that do not accumulate ER bodies, and are suppressed in whole tissues by the introduction of the *nai1* mutation, suggesting a strong relationship between ERMO3 and ER bodies. The ER body structure is not significantly affected in *ermo3* cells (Marti et al., 2010; Nakano et al., 2012), and ERMO3 does not interact with NAI2 (Nakano et al., 2012), suggesting that the contribution of ERMO3 to ER body formation is not highly significant. ERMO3 was required for proper protein transport between ER and Golgi bodies, and ERMO3 formed a protein complex with PYK10, JAL, and MATH domain-containing proteins, which are regulated by NAI1 (Nakano et al., 2012). These results suggest that PYK10, JAL, and MATH domain-containing proteins form a large protein aggregate on ER, then alter the ER morphology to inhibit protein secretion in the absence of ERMO3. ERMO3 may be responsible for solving this aggregate in ER, because the expression of ERMO3 in ER rescues the phenotype in the *ermo3* mutant (Nakano et al., 2012). This tissue-specific requirement of ERMO3, in addition to the ubiquitous expression of ERMO3, clearly shows that each cell type requires its own regulatory systems to maintain their subcellular organization.

## Physiological functions of ER bodies: putative association with innate immunity

### Tissue specificity and wounding inducibility indicate the importance of ER body in plant defense

**Figure 3:**
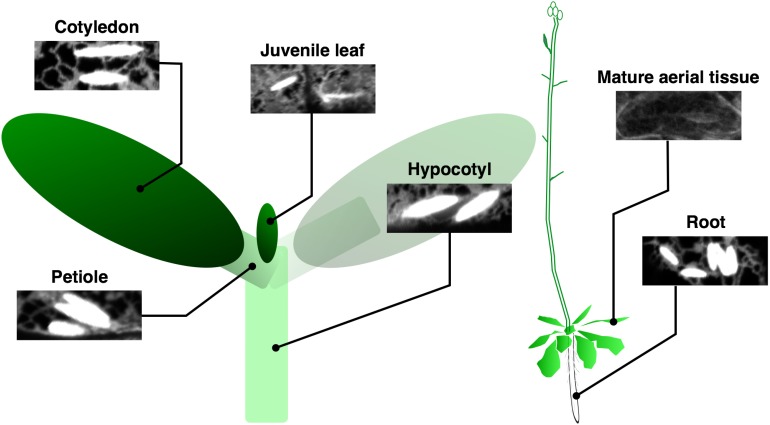
**Tissue localization of ER bodies**. ER bodies are present in the epidermis of whole tissues in young seedlings, but are absent in aerial organs of mature plants. Root epidermal, cortical, and endodermal cells continue to develop ER bodies. Epidermal cells of a transgenic *A. thaliana* expressing ER-localized GFP from several tissues are shown.

Constitutive ER bodies in *A. thaliana* are enriched in seedlings and roots of mature plants (Matsushima et al., 2002). In cotyledons, ER bodies develop only in epidermal cells but not in mesophyll cells. Similarly, no ER bodies are detected in the root vascular cylinder, in contrast to their presence in root epidermal, cortical, and endodermal layers (Figure 3). Shoot tissues including rosette and cauline leaves have fewer ER bodies compared to those of the underground tissues. They are absent in most of the rosette leaf cells, except for some epidermal cells along the primary and secondary veins or the edge of the leaves (Nakano et al., 2012). These specificities among plant tissues and cell types suggest that ER bodies are enriched at the interface between plants and surrounding organisms to protect plants from pathogens/herbivores that may enter or feed from the veins or leaf edge. This is supported by the fact that leaf wounding triggers local and systemic *de novo* formation of ER bodies in a jasmonic acid (JA)-dependent manner (Matsushima et al., 2002; Ogasawara et al., 2009). Atypical, elongated ER bodies are produced after wounding in the *nai1* mutant, suggesting that another factor in addition to NAI1 is involved in this response (Matsushima et al., 2003). Consistent with this, the induction of BGLU18, a major component of wound-triggered ER bodies in rosette leaves, is independent of NAI1 (Ogasawara et al., 2009). The observed high abundance of ER bodies in roots may contribute to the interaction between plants and surrounding (potential) pathogens and herbivores that inhabit soil (Yamada et al., 2011). For example, the soil environment contains 10^6^–10^9^ bacteria per gram, which is much greater than in the atmosphere (10^1^–10^5^ per cubic meter) (Bulgarelli et al., 2013). Many other organisms such as insects, worms, nematodes, and fungi also live in the soil and seek opportunities to exploit plant roots.

### β-glucosidase activity of PYK10 may contribute to the plant immune response

The high abundance of PYK10 and other β-glucosidases in ER bodies implies that the enzymatic activities of these proteins play an important role in the function of this organelle. The reported *in vitro* substrates of PYK10 include 4-methylumbelliferyl (4MU)-glucoside, 4MU-fucoside (Matsushima et al., 2004), scopolin, and esculin (Ahn et al., 2010). Recombinant PYK10 protein expressed in insect cells hydrolysed with the highest efficiency scopolin (Ahn et al., 2010), a coumarin widely occurring in the plant kingdom, including *A. thaliana* (Bednarek et al., 2005; Shimizu et al., 2005; Kai et al., 2006; Bayoumi et al., 2008). Scopolin is a β-*O*-glucoside of scopoletin, which is regarded as a phytoalexin. It was shown that scopoletin and scopolin could inhibit germ tube growth of *Sclerotinia sclerotiorum* (Prats et al., 2006). Scopoletin was also found to possess toxic activity against *Fusarium oxysporum, Fusarium solani, Rhizopus stolonifer*, and *Lasiodiplodia theobromae* (Peterson et al., 2003). Growth inhibition of *F. oxysporum* was higher with scopoletin than with scopolin, suggesting that a β-glucosidase could have an important role in this activity.

Concerning the biological activity of scopoletin, it is possible that ER bodies are involved in plant defense by hydrolysing scopolin to this aglycone. As discussed above, JAL proteins, GLL proteins, and MATH domain-containing proteins formed a large protein complex with PYK10 after cell disruption (Nagano et al., 2005, 2008; Ahn et al., 2010). These proteins accumulate constitutively in separate cellular organelles: PYK10 in ER body, GLLs in vacuole, JALs in cytosol, and MATHs at plasma membrane (Nagano et al., 2005; Oelmuller et al., 2005; Marti et al., 2010; Nakano et al., 2012). The β-glucosidase activity of PYK10 is higher after complex formation, suggesting that JALs and GLLs serve as activators of PYK10 (Nagano et al., 2005, 2008). Stimulation of β-glucosidase activity can be solely carried out by PBP1, as revealed by an *in vitro* enzymatic assay (Ahn et al., 2010). This fact, combined with wound inducibility, suggests that these proteins are assembled with PYK10 when cells are damaged by feeding insects or pathogen infection, and that they produce substances that potentially target these invaders.

### PYK10 may have myrosinase activity toward indole glucosinolates

β-Glucosidases in the order *Brassicales* include a unique class of enzymes named myrosinases or β-thioglucoside glucohydrolases (TGGs), which are involved in the defense against insects, fungi, and bacteria (reviewed in Hopkins et al., 2009). Myrosinases are responsible for hydrolysing glucosinolates, thioglucosides that are specific to the order *Brassicales* (Halkier and Gershenzon, 2006). For a long time, myrosinases were considered to contain a unique amino acid signature that enables *in silico* prediction of their identity based on nucleotide sequence data (Burmeister et al., 1997). One of the unique features of this signature is a conserved basic residue (lysine or arginine) at the substrate pocket, which can form electrostatic interactions with the negatively charged sulfate group of glucosinolates (see Figure 5). It was found that the glutamate residue that serves as a proton donor in *O*-glucosidases is substituted by glutamine in myrosinases, resulting in the strict reduction of *O*-glucosidase activity (Burmeister et al., 1997). In *A. thaliana*, six genes (*TGG1−6*) including two pseudogenes [*TGG3*, (Zhang et al., 2002); *TGG6*, (Andersson et al., 2009)] encode myrosinases. TGG1 and TGG2 are expressed primarily in leaves (Xue et al., 1995), whereas TGG4 and TGG5 are expressed primarily in roots (Barth and Jander, 2006; Andersson et al., 2009; Zhou et al., 2012). These four myrosinase have conserved lysine/arginine and glutamine residues at the substrate pocket, and have activity against glucosinolates. By contrast, the other β-glucosidases lacking these specific amino acids were thought to be β-glucosidases hydrolysing *O*-glucosides but not thioglucosides (Rask et al., 2000). However, PEN2, a β-glucosidase lacking these key amino acids, was recently shown to be an atypical myrosinase hydrolysing indol-3-ylmethyl glucosinolate (I3G) and 4-methoxy-I3G (4M-I3G) (Bednarek et al., 2009). PEN2 has a major role in *A. thaliana* immunity via its myrosinase activity (reviewed in Bednarek, 2012) against various microbes including fungal pathogens such as *Blumeria graminis* and *Plectosphaerella cucumerina* (Sanchez-Vallet et al., 2010), *Magnaporthe oryzae* (Maeda et al., 2009), *Leptosphaeria maculans* (Elliott et al., 2008), *Colletotrichum* species (Hiruma et al., 2010), oomycetes [e.g., *Phytophthora brassicae* (Schlaeppi et al., 2010) and *Pythium irregularum* (Adie et al., 2007)], and a growth-promoting endophytic fungus (*Piriformospora indica*; Jacobs et al., 2011).

**Figure 4:**
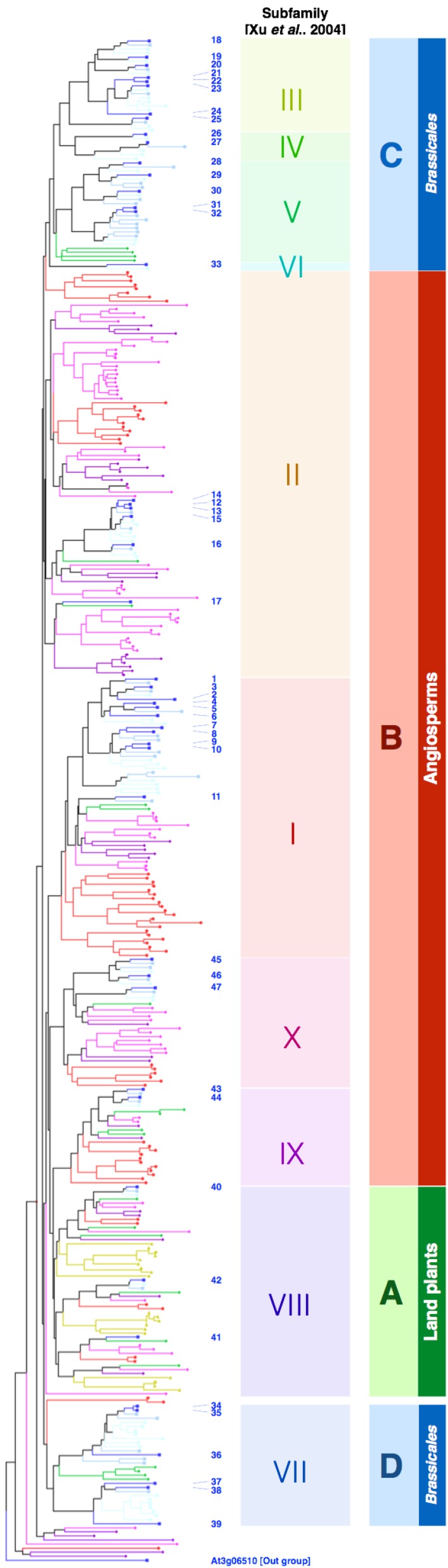
**Both classical and atypical myrosinases form *Brassicales*-specific clades**. The full-length protein sequences of β-glucosidases in *A. thaliana* (blue squares), *Capusella rubella* (light-blue squares), *Thellungiela salsuginea* (cyan squares), *Carica papaya* (green diamonds), *Vitis vinifera* (dark purple diamonds), *Glycine max* (magenta diamonds), *Oryza sativa* (red circles), and *Physcomitrella patens* (dark yellow triangles) were retrieved from the Phytozome database (http://www.phytozome.net/) and aligned by ClustalW (http://clustalw.ddbj.nig.ac.jp/index.php). BGLU numbers of the β-glucosidases in *A. thaliana* are indicated by blue letters. At3g06510 was used as an out group. Subfamilies proposed by Xu et al. (2004) are indicated accordingly.

**Figure 5:**
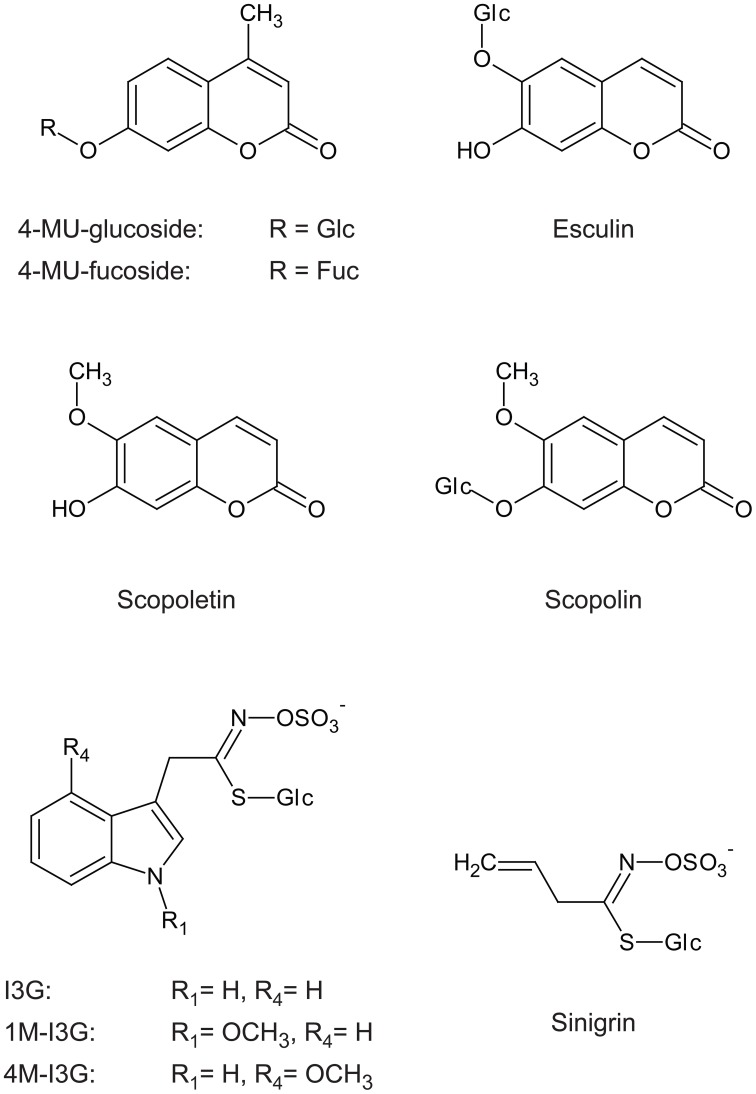
**Indole glucosinolate structure includes a side chain similar to that of scopolin and esculin, but not similar to that of sinigrin**. I3G, indol-3-ylmethylglucosinolate; 1M-I3G, 1-methoxyindol-3-ylmethylglucosino late; 4M-I3G, 4-methoxyindol-3-ylmethylglucosinolate; 4-MU-glucoside, 4-methylumbelliferyl-β-D-glucoside; 4-MU-fucoside, 4-methylumbelliferyl-β-D-fucoside.

PEN2 does not belong to the subfamily of PYK10, but both proteins have high sequence similarity (Figure 4), which suggests that PYK10 also has atypical myrosinase activity, whereas recombinant PYK10 was unable to hydrolyse sinigrin (Ahn et al., 2010). Sinigrin, which represents aliphatic glucosinolates, consists of the glucosinolate core structure with a short aliphatic side chain that differs significantly in its structure from the indolyl group present in I3G and 4M-I3G (indole glucosinolates) (Figure 5). It cannot be excluded that PYK10 may hydrolyse indole glucosinolates in addition to its inactivity toward aliphatic glucosinolates. The reported substrates for PYK10 have aglycones of coumarinyl moieties, suggesting that the substrate pocket of PYK10 is suitable for condensed-ring moieties. This also supports the possible activity of PYK10 to hydrolyse indole glucosinolates, which have condensed-ring moieties as aglycones (Figure 5). According to Brown et al. (2003), indole glucosinolates are the most abundant glucosinolates in *A. thaliana* roots (at least of Col-0 accession), where ER bodies and PYK10 are also highly abundant. Coexpression analysis based on a public microarray database (ATTED-II; http://atted.jp) revealed a strong correlation between *PYK10* and *CYP81F4* encoding an enzyme involved in the modification of I3G into 1-methoxy-I3G (Pfalz et al., 2011), further suggesting a functional link between PYK10 and indole glucosinolates.

### PYK10 in mutualistic relationship with *Piriformospora indica* and defense against parasitic nematodes

It was reported that transgenic *A. thaliana*, which produced a novel type of glucosinolates, showed distinct bacterial and fungal communities in its rhizosphere compared to those of WT plants (Bressan et al., 2009). This result strongly suggests the involvement of glucosinolate in pathogenic and mutualistic plant microbe interactions in the soil environment. One of the most extensively studied root-colonizing microorganisms is an endophytic fungus *P. indica* that has a broad range of host species and a capacity to promote host-plant growth (Varma et al., 1999). Similar to the *pen2* mutant, *pyk10* and *nai1* mutant plants failed to suppress *P. indica* growth at a preferable level, resulting in fungal overgrowth that stimulated undesirable immune responses and loss of the beneficial interaction (Sherameti et al., 2008; Jacobs et al., 2011). In addition, depletion of two cytochrome P450 enzymes (CYP79B2 and CYP79B3) that are responsible for conversion of tryptophan into indole-3-acetaldoxime (IAOx), the first step of the biosynthesis of indole glucosinolates, also resulted in overgrowth of *P. indica* (Nongbri et al., 2012). By contrast, the phytoalexin camalexin, which is derived from IAOx and is absent in the *cyp79B2 cyp79B3* mutant, was dispensable for this suppression, suggesting that the proper control of this mutualistic fungus may involve indole glucosinolate metabolism. These results suggest that PYK10, PEN2, and indole glucosinolates have important roles in the establishment of the beneficial interaction with *P. indica*.

*PYK10* was originally isolated as a root-specific gene with high expression level that became additionally elevated in nematode-infected tissue (Nitz et al., 2001). Because the degradation products of glucosinolates exhibited inhibitory activity against both a cyst nematode (*Globodera rostochiensis*; Buskov et al., 2002) and a root-knot nematode (*Meloidogyne incognita*; Lazzeri et al., 2004), it is possible that PYK10 protects roots from these parasites by its potential myrosinase activity. Cyst nematodes degrade the cell wall and infect *intracellularly*, whereas root-knot nematodes infect *intercellularly* (reviewed in Mitchum et al., 2012). In both cases, the cells surrounding infectious nematodes are disrupted and possibly collapse, which can passively trigger hydrolysis of glucosinolates. Alternatively, the plant may recognize the presence of these pests via nematode-associated molecular patterns or effectors injected into plant cells, and actively exert defensive glucosinolate metabolism.

Although both *pen2* and *pyk10* mutants showed greater *P. indica* colonization (Sherameti et al., 2008; Jacobs et al., 2011), there was no direct evidence that the control of fungal growth during this interaction was due to the myrosinase activity of both enzymes toward indole glucosinolates. Similarly, in addition to the inhibitory activity of glucosinolates against parasitic nematodes, it is not known if myrosinases are involved in these interactions. In addition to PEN2 and PYK10, *A. thaliana* roots express two classical myrosinases (TGG4 and TGG5), and TGG4 was shown to metabolize indole glucosinolates when overexpressed *in planta* (Bednarek et al., 2009). Future work will determine which of these four enzymes, and possibly other atypical myrosinases, are the key players during the interaction with endophytic fungi and parasitic nematodes.

### Enhanced secretion of proteins localized in ER bodies (ESPER) is a novel immune response against pathogens

Recently, Watanabe et al. (2013b) reported a novel immune response designated as ESPER. They showed that two plant pathogenesis-related (PR) proteins, defensin (PDF1.2) and PR1, which contain antimicrobial activity, accumulated in ER bodies at a steady-state level and were secreted to the apoplast in response to the non-adapted pathogenic fungi *Colletotrichum gloeosporioides*. This suggests that ER bodies can serve as storage sites for various PR proteins until pathogenic attack, although these proteins have been reported to localize primarily in the vacuole or apoplast (Keefe et al., 1990; Dixon et al., 1991; Neuhaus et al., 1991; Van Loon et al., 2006; Sels et al., 2008). However, ER bodies can be labeled with GFP fused to proteins in the secretory pathway, including both soluble and membrane proteins, which finally accumulate in other organelles (Teh and Moore, 2007), suggesting the non-selective entry of these proteins into ER bodies. The localization of PDF1.2 and other PR proteins was analyzed using GFP-fused protein constructs driven by the artificial cauliflower mosaic virus (CaMV) 35S promoter (Watanabe et al., 2013b). Therefore, it is possible that the observed protein accumulation in ER bodies could have been an artefact due to gene overexpression. Nevertheless, the drastic change in localization of these GFP-fused PR proteins under fungal attack indicates that the membrane trafficking system in plant cells can dynamically change in response to the surrounding environment. Although the ER body itself is not involved in ESPER, as suggested by the presence of ER bodies even after ESPER occurred, this, in turn, suggests that localization of PYK10 and other ER body components may also dynamically respond to abiotic/biotic conditions to become active.

### Potential ER body function in metal tolerance or uptake

**Figure 6:**
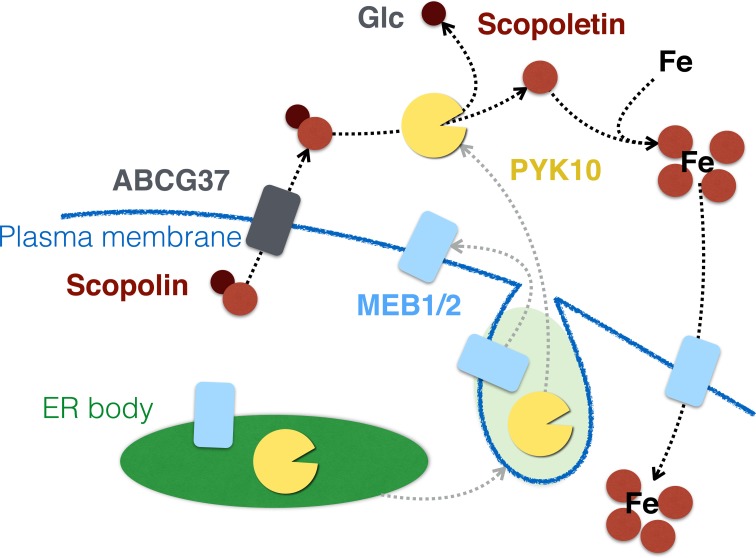
**PYK10 and ER bodies may have a role in iron uptake via hydrolysing scopolin**. ER bodies may fuse with the plasma membrane under iron-deficient conditions, resulting in the relocation of MEB1 and MEB2 to the plasma membrane and secretion of PYK10 to the apoplast. Scopolin, secreted in an ABCG37-dependent manner, is then converted to scopoletin by PYK10, which in turn helps the cells to take up iron via the chelating activity of scopoletin. This uptake may be carried out by putative MEB transporters.

Recently, it was reported that iron starvation stimulated an ATP-binding cassette transporter ABCG37 (PDR9)-dependent secretion of scopoletin (Fourcroy et al., 2014). Roots of the *pdr9* mutants that lacked functional ABCG37 failed to secrete scopoletin and showed abnormal accumulation of its derivatives, including scopolin, inside the tissue. These mutants also exhibited decreased tolerance to iron deficiency, suggesting that secretion of scopoletin helped plants to take up iron. This is further supported by the results that some of the coumarin derivatives have the potential to chelate iron and serve as phytosiderophores (Mladenka et al., 2010). Therefore, scopoletin, produced by the action of PYK10 on scopolin, may contribute to iron uptake in *A. thaliana* roots. Notably, MEB1 and MEB2, the ER body-specific membrane proteins, were recently suggested to be iron transporters that can endow *S. cerevisiae* with iron tolerance when expressed heterologously (Yamada et al., 2013). These results suggest that ER bodies have a role in metal-stress response apart from plant microbe interactions. One possibility is that under iron-limiting conditions, ER bodies fuse with plasma membrane to release PYK10 into the apoplast, which consequently hydrolyses scopolin that is secreted to the apoplast via ABCG37 (Figure 6). Liberated scopoletin may enhance the availability of iron, which can be taken up via MEB putative transporters that are transferred on the plasma membrane. Mugineic acid, a phytosiderophore in barley, accumulates in ER-derived vesicles peripheral to the plasma membrane of root epidermal cells, and can be secreted to the soil to improve the iron availability (Negishi et al., 2002). To date, there is no report elucidating how ER bodies behave in iron-depleted conditions. In addition, it should be noted that the experiments reported by Fourcroy et al. (2014) were performed with roots of plants grown on agar plates and exposed to light. The accumulation of phenylpropanoids (the group of metabolites that includes scopolin and scopoletin) is strongly upregulated in *A. thaliana* roots artificially exposed to light, as compared to roots grown in soil (Hemm et al., 2004), suggesting that in soil the scopoletin-dependent iron-uptake mechanism may have reduced significance. Therefore, it is of great interest to test ER body behavior, together with scopoletin production, in *A. thaliana* roots grown in iron-depleted soil, which may validate the function of ER bodies in metal tolerance or uptake.

### Stress translation via phytohormone activation

BGLU18/AtBG1 and BGLU33/AtBG2, which belong to the same clade with PYK10 and PEN2 (see Figure 4, discussed below), were reported to be involved in abscisic acid (ABA) signaling and drought resistance by hydrolysing ABA-*O*-glucoside, which is an inactive pool of ABA (Lee et al., 2006; Han et al., 2012; Xu et al., 2012; Watanabe et al., 2013a). BGLU18 is a member of the PYK10 subfamily. According to the ATTED-II database, BGLU18 is expressed primarily in aerial organs such as leaves and stems, but is limited in roots, which negatively correlates with the presence of constitutive ER bodies. However, BGLU18 is a component of ER bodies that are induced by wounding (Ogasawara et al., 2009). *BGLU18* is co-expressed with some homologs of genes related to constitutive ER bodies including *TSA1*, the closest homolog of *NAI2, JAL* genes (*JAL23* and *JAL35*), and a *GLL* gene, strongly suggesting that the mechanisms of BGLU18 accumulation and activation could be similar to those reported for PYK10.

Presuming that ABA is activated through sugar release from ABA-glucoside in drought-stressed leaves, it could be speculated that BGLU18 is engaged in ABA-activation in a manner independent from both constitutive root ER bodies and wound-inducible leaf ER bodies. Because the dynamics of ER bodies under drought stress are still unclear, it is possible that these organelles are induced under water-deficit conditions to mediate the conversion of ABA-*O*-glucoside to ABA, in response to the signal transduction of drought stresses. According to such a scenario, BGLU18 expression is induced by wounding and by drought stress (ATTED-II), suggesting its dual functions in stress pathways. BGLU19, another member of the PYK10 subfamily, was suggested to be involved in cytokinin activation based on the highest sequence similarity with the β-glucosidase of *Brassica napus* that hydrolyses zeatin-*O*-glucoside (Falk et al., 1995; Xu et al., 2004). These results suggest that some β-glucosidases from this clade may have potential roles in phytohormone activation. The accumulation of various glucosylated phytohormones as precursors (Gachon et al., 2005; Poppenberger et al., 2005; Lee et al., 2006) suggests the involvement of glucosidases in the activation of these compounds, where the β-glucosidases may participate. Elucidating the involvement and behavior of ER bodies in phytohormone-mediated stress responses is required to further understand the functions of this organelle.

## Evolution of ER bodies and β-glucosidases in the order *Brassicales*

### ER bodies are formed in plants belonging to the *Brassicales* order

ER bodies were frequently observed by electron microscope using root tissues of plants belonging to the order *Brassicales* (Bonnett and Newcomb, 1965; Iversen and Flood, 1969; Iversen, 1970a; Cresti et al., 1974; Hoefert, 1975; Endress and Sjolund, 1976; Jørgensen et al., 1977; Behnke and Eschlbeck, 1978; Gailhofer et al., 1979; Jørgensen, 1981). This order includes *Brassicaceae* (mustard and cabbage family, including *A. thaliana*), *Capparaceae* (caper family), *Cleomaceae* (cleome family), *Resedaceae* (mignonette family) and *Tovariaceae*. The plants belonging to *Brassicaceae, Cleomaceae*, and *Capparaceae* are closely related to each other (Hall et al., 2004), and develop ER bodies or ER body-like structures in stems or roots. The ER bodies in these species occasionally contain internal filamentous structures (Hoefert, 1975; Jørgensen et al., 1977; Behnke and Eschlbeck, 1978; Gailhofer et al., 1979), suggesting their well-ordered formation. To date, no ER bodies have been observed in the *Resedaceae* (Iversen, 1970a). The *Tovariaceae* also do not develop ER bodies; however, filamentous aggregations of electron-dense materials similar to that of ER body were observed in the vacuoles of vascular-bundle cells (Behnke and Eschlbeck, 1978). Considering that *Tovariaceae* separated from *Brassicaceae* approximately 40−50 million years ago (Martin-Bravo et al., 2009), these filamentous structures might be the evolutionary intermediates of ER bodies. Collectively, ER bodies and related structures develop exclusively in a taxonomically limited group, raising the question of how this organelle evolved in these families.

The plant order *Brassicales* includes agriculturally important crops such as *Brassica rapa* and the model species *A. thaliana*, and plants in this order have been studied intensely. Many *Brassicales* plants are characterized with some unique features that evolved in this order, including the production of glucosinolates and other sulfur-containing secondary metabolites (Mithen et al., 2010; Bednarek et al., 2011), and the inability to accept arbuscular mycorrhiza as symbionts (reviewed in Delaux et al., 2013). Based on the public genome sequences, most of the proteins that localize in ER bodies and those that participate in the PYK10 protein complex belong to clades that occur in the family *Brassicaceae*. This suggests that *A. thaliana*, and most likely other species in *Brassicales*, have innovated a set of specific genes for developing these unique organelles.

### β-glucosidases of the PYK10 subfamily evolved in the *Brassicales* order

The *A. thaliana* genome encodes 47 β-glucosidases, which are grouped into ten subfamilies (Xu et al., 2004). Figure 4 shows a neighbor-joining phylogenic tree generated for the *A. thaliana* β-glucosidases and related proteins retrieved from the genome sequences of *Capsela rubella, Tellungiella salsuginea* (formerly *T. halophila*), *Carica papaya, Vitis vinifera, Glycine max, Oryza sativa*, and *Physcomitrella patens*. The clade A includes BGLU40, 41, and 42, which corresponds to subfamily 8 (shaded with green); it also includes the β-glucosidases from *P. patens*, suggesting that these clades are ancestral among land plants. Clade B (shaded with red) corresponds to subfamilies 1, 2, 9, and 10; it does not include enzymes from *P. patens*, but does include enzymes from all other species, suggesting that this clade is specific to the angiosperms. By contrast, clades C and D correspond to the subfamilies 3–6, 7, and appear to be specific to *Brassicales*. Clade D consists of myrosinases, which are known to be specific for this order. The β-glucosidases from *C. papaya* were excluded from the subfamilies 3–5 that contain PYK10 and PEN2, showing the specificity of these three subfamilies to *Brassicaceae*, and possibly other species between *Brassicaceae* and *Caricaceae*. We identified a different lysine/arginine residue from the one conserved in classical myrosinases, which is located at the surface of the substrate pocket and completely conserved in all members of clade C (unpublished data). This positively charged residue might form electrostatic interactions with glucosinolates and explain the myrosinase activity of PEN2. Furthermore, it suggests that other β-glucosidases grouped into the clade are atypical myrosinases. As the glutamate proton donor residue is conserved within clade C, these enzymes appear to be intermediate forms between typical β-*O*-glucosidases and classical myrosinases. Twenty-two β-glucosidases in *A. thaliana* belong to clades C and D (hereafter called EE[K/R]-type and QE[K/R]-type, respectively), which is approximately half of the total β-glucosidases. Although myrosinases have been proposed to be special β-glucosidases, our phylogenetic analyses suggest that myrosinase activity could be a rather common feature of the β-glucosidases in *Brassicales*. The wide diversity within these β-glucosidases suggests that they have substrate specificity toward specific glucosides that are different in each plant tissue or under distinct conditions. This differential specificity would fit with glucosinolates that are highly diversified within *Brassicales* (reviewed in Agerbirk and Olsen, 2012).

It is unknown whether QE[K/R]-type β-glucosidases (classical myrosinases) evolved from EE[K/R]-type β-glucosidases (atypical myrosinases), or if they emerged independently. Two myrosinases that were recently isolated from *Carica papaya* possess the QE signature; however, they do not have any basic [K/R] residues that may mediate electrostatic interactions with glucosinolates. Consequently, both enzymes have a lower affinity for sinigrin compared to that of the classical myrosinases in *A. thaliana* (Nong et al., 2010). *C. papaya* is located almost at the edge of the *Brassicales* phylogenic tree (Hall et al., 2004), and shared a common ancestor with *A. thaliana* approximately 72 million years ago (Ming et al., 2008), suggesting that myrosinases evolved from these ancient forms that lacked the glucosinolate-binding affinity. Because glucosinolates have been identified in *C. papaya* (Tang, 1973), evolution of this basic residue [K/R] might have been achieved after the innovation of glucosinolates. The specific positions of these conserved basic residues differ between classical and atypical myrosinases; thus, it is likely that these two groups emerged independently. According to the public genome database, the *C. papaya* genome does not encode a protein bearing significant similarity to NAI2 from *A. thaliana*, whereas other species in *Brassicaceae* do, suggesting that ER bodies have evolved specifically in plants belonging to the *Brassicaceae*, possibly due to the evolution of *NAI2*. The evolutionary process of producing ER bodies and atypical myrosinases will be elucidated by analysing species in between *C. papaya* and *Brassicaceae*.

### Plants of the *Brassicales* order develop distinct glucosidase-glucoside defense systems

**Figure 7:**
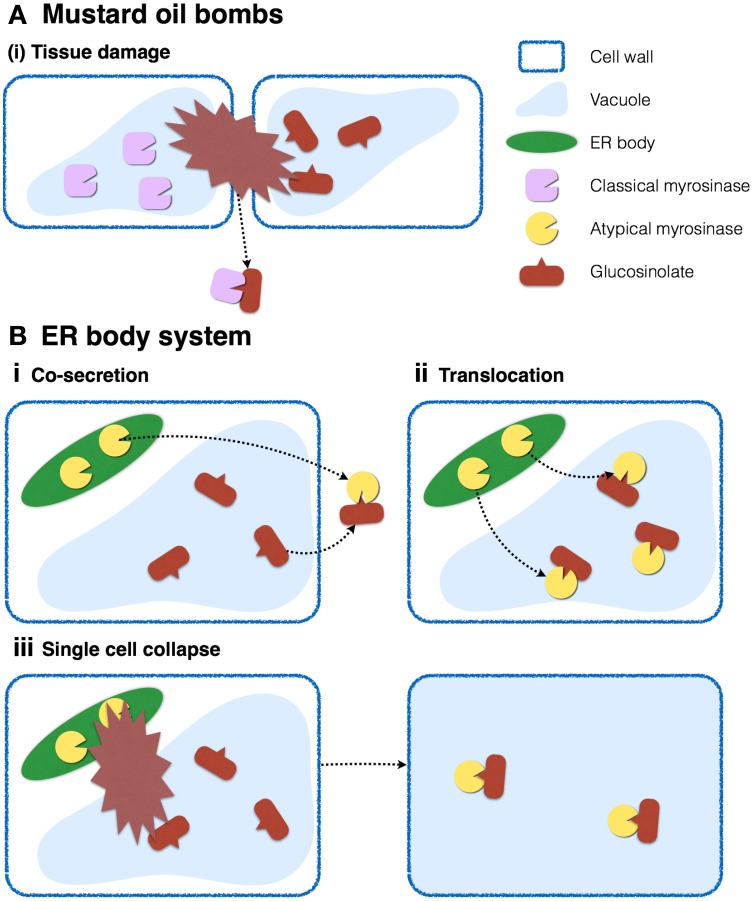
**Two distinct myrosinase-glucosinolate systems occurring in *Brassicales***. **(A)** In leaves, myrosinases and glucosinolates accumulate in vacuoles of distinct cells. Tissue collapse accompanied by destruction of multiple cells results in the mixing of enzymes and substrates, and the production of toxic products against herbivores. **(B)** In roots, myrosinases and glucosinolates accumulate in ER bodies and vacuoles, respectively, in the same cells. Enzymatic reactions may be activated by **(i)** cosecretion of enzymes and substrates, **(ii)** translocation of enzymes into vacuoles, or **(iii)** destruction of a single cell.

To avoid constitutive hydrolysis of glucosinolates, which are proposed to accumulate in vacuoles, myrosinases have to be separated from their substrates into distinct cellular or subcellular compartments (Figure 7). In the case of classical myrosinases, the mustard-oil bomb system was proposed, in which the enzymes and substrates accumulate in distinct cells called myrosine cells and S cells, respectively, which allows suppression of the enzymatic reaction until the tissue is crushed. This intercellular partitioning does not require specific subcellular compartments for storage, and myrosinases accumulate in the vacuoles of myrosine cells possibly because they have the largest volume.

Table 1 shows that many β-glucosidases contain signal peptides, but some of them lost this motif. Differential localization might indicate that β-glucosidase activities are required for various cellular functions including those of endomembrane systems, apoplast, and cytosol. The evolutionary events of losing signal peptides appear to occur independently, suggesting that each of these events could reflect the acquisition of a novel molecular function. For example, PEN2 is known to localize at the periphery of peroxisomal membranes, which enables it to accumulate at the penetration sites in response to *Blumeria graminis* f. sp. *hordei* (*Bgh*) challenge (Lipka et al., 2005). This clearly shows the importance of protein localization for protein function. The mechanism of glucosinolate activation by PEN2 is totally different from that of classical myrosinases, because *Bgh* challenge does not inflict cell damage or cell disruption.

It is possible that *Brassicales* plants evolved ER bodies to store atypical myrosinases and separate them from their substrates on the subcellular level. A recent study addressing the cell-type specific metabolome in *A. thaliana* roots revealed the cellular distribution of glucosinolates (Moussaieff et al., 2013). According to this study, indole glucosinolates accumulate to relatively higher levels in columella and cortex, whereas lower levels accumulate in epidermis, endodermis, and stele, indicating that PYK10 and indole glucosinolates occur concurrently in the same cells in the cortex of *A. thaliana* roots, but in different organelles. ER bodies contain PYK10, whereas vacuoles presumably store indole glucosinolates, suggesting that plants in the *Brassicales* order evolved two distinct myrosinase-glucosinolate systems, one of which is the classical mustard-oil bomb system described above. The system proposed here considers that ER bodies separate both components into distinct organelles in a single cell (Figure 7B). This subcellular compartmentalization, which does not require tissue collapse to trigger the response, could be highly effective against fungal/bacterial pathogens that usually start the infection process by invading single plant cells. This system could be triggered by translocation of enzymes and/or substrates in living cells, such as the case of PEN2 (Lipka et al., 2005; Bednarek et al., 2009). It is also possible that chemical or protein factors such as pH or chaperones regulate the activity of ER body-accumulating β-glucosidases.

According to the literature, glucosinolates and myrosine cells are widely distributed throughout the *Brassicales* (Jørgensen, 1981). By contrast, ER bodies showed higher taxonomic limitation primarily in *Brassicaceae, Cleomaceae*, and *Cappraceae* (Iversen, 1970a; Behnke and Eschlbeck, 1978; Jørgensen, 1981), suggesting that ER bodies are a more recent innovation among these families. Due to the lack of genetic/molecular tools in species belonging to other *Brassicales* families than *Brassicaceae*, the evolution of specific substrates, enzymes, and organelles remains unclear. Recent improvements in sequencing technologies have facilitated rapid progress in genome sequences for non-model plants, including many *Brassicaceae* species such as *T. salsuginea* (Wu et al., 2012; Yang et al., 2013), *B. rapa* (Wang et al., 2011), *C. rubella* (Slotte et al., 2013), and *A. lyrata* (Hu et al., 2011). Molecular and genetic tools are becoming available that will enable the inclusion of many other species in future research.

## Concluding remarks and future perspectives

We have shown how ER bodies are unique in their shape, biogenesis mechanism, functions, and evolution. The study of ER bodies provides novel insights into plant innate immunity systems, stress tolerance, and the subcellular organization of eukaryotic cells. Cell biological studies for understanding eukaryotic organelle organization are sometimes too focused on single cell types, including cultured cells. However, cultured cells are very different from endogenous cells in living individuals. For example, tobacco BY-2 cells are one of the most widely utilized plant cultured cell lines, but they have ER structures that are slightly different to those in *A. thaliana*, lacking ER bodies, and tubules and cisternae that appear different (Nakano et al., 2009). It is necessary to keep in mind what cell types are used for the experiments, and to clearly distinguish what is unique to the cell type and what is common to all eukaryotic cells, or at least to the species in use.

The ER body is a microdomain of the ER. Numerous studies have focused on how microdomains in the plasma membrane are organized and contribute to cell functions (reviewed in Lingwood and Simons, 2010). These membrane microdomains also play important roles in plant cells (reviewed in Malinsky et al., 2013). However, because these studies focused primarily on microdomains in plasma membranes, little is known about the organization of microdomains in organelle membranes including the ER. The organization of lipids and proteins should play an important role in the maintenance of organelle morphology and function. ER bodies may be a good model to understand microdomain organization in ER. The mechanisms underlying ER body formation will provide valuable insights into the general mechanisms of microdomain organization for organelle membranes. Combined with an understanding of physiological functions, ER bodies can be an interesting model case to connect subcellular microdomains to the overall fitness of individual plants.

Yet, we still have some unanswered key questions. The most important piece of the puzzle that is missing is the genetic evidence for ER body function in stress responses. As discussed in the second section, most results achieved in this decade suggest a role of ER bodies in interaction with microbes and in abiotic-stress responses via activation of phytohormones and/or phytosiderophores. However, there is still no direct evidence for the physiological functions of ER bodies. Identification of the native substrate(s) could help us in validating the function(s). Phenotypic analysis of knockout lines deficient either in ER-bodies, PYK10 or substrates of this β-glucosidase, as well as respective multiple mutants subjected to various conditions will be required toward this end. To confirm the involvement of PYK10 and its substrate in the response to a certain environmental stress it will be necessary to show that the mutants depleted in either the enzyme or the substrate show a similar defect at least in regard to that particular response. More importantly, the multiple mutant lacking both the enzyme and the substrate should exhibit comparable, but not additive or synergistic phenotype compared to the mutants lacking only the substrates or the enzymes. Performing such genetic analysis, particularly when both enzymes and substrates are involved in multiple pathways, will provide insights into which combination of enzymes and substrates is registered to the pathway of interest. What is also completely missing is the behavior of ER bodies in response to these stresses; thus understanding the dynamics of ER bodies and PYK10 is crucial. The proper response to stress requires activation of molecules that are involved in the resistance to stress, e.g., toxic compounds should be produced only when plants are subjected to undesirable visitors, because these compounds may also be toxic to the plant. This responsive activation may include dynamic translocation and/or secretion of enzymes, where the ER body β-glucosidases can become activated (Figure 7).

The advantage or disadvantage of developing specific storage organelles is not fully understood. Many plant species evolved specific organelles for protein storage (reviewed in Hara-Nishimura and Matsushima, 2003). The production of special compartments or compounds does require valuable resources, as shown for glucosinolates (Bekaert et al., 2012; Züst et al., 2012), which suggests that the possession of ER bodies should confer a beneficial advantage for plant fitness. To specifically address the significance of accumulating PYK10 in ER bodies, a population genetics approach using generations of *nai1, pyk10*, and *nai2* mutants under appropriate conditions will be required. Revealing the evolutionary processes during the achievement of ER bodies will also provide insight into this issue. Toward this end, a genome sequence of a species in between *Brassicaceae* and *Caricaceae* will be a great help.

### Conflict of interest statement

The authors declare that the research was conducted in the absence of any commercial or financial relationships that could be construed as a potential conflict of interest.
